# Quality of life and olfactory function after suprasellar craniopharyngioma surgery—a single-center experience comparing transcranial and endoscopic endonasal approaches

**DOI:** 10.1007/s10143-020-01343-x

**Published:** 2020-07-10

**Authors:** Sascha Marx, Ioanna Tsavdaridou, Sebastian Paul, Antje Steveling, Cornelia Schirmer, Marton Eördögh, Stephan Nowak, Marc Matthes, Ehab El Refaee, Steffen K. Fleck, Joerg Baldauf, Markus M. Lerch, Andreas Stahl, Werner Hosemann, Henry W. S. Schroeder

**Affiliations:** 1 Department of Neurosurgery, University Medicine Greifswald, Sauerbruchstraße, 17475 Greifswald, Germany; 2 Department of ENT, University Medicine Greifswald, Greifswald, Germany; 3 Department of Ophthalmology, University Medicine Greifswald, Greifswald, Germany; 4 Department of Internal Medicine, University Medicine Greifswald, Greifswald, Germany; 5 Department of Neurosurgery, Cairo University, Giza, Egypt

**Keywords:** Craniopharyngioma, Skull base tumors, Quality of life, Olfactory function, Long-term follow-up, Endonasal approach, Transcranial approach

## Abstract

The endoscopic endonasal approach to suprasellar craniopharyngiomas has become popular as alternative to transcranial approaches. However, the literature lacks data regarding quality of life and olfactory function. The assessment of the long-term quality of life and olfactory function of all patients harboring a suprasellar craniopharyngioma who underwent surgery in our department has been done. Patient characteristics and perioperative data were gathered in a prospectively maintained database. At the last follow-up visit, the olfactory function and the quality of life (ASBQ, SNOT-22) as well as visual and pituitary function were assessed. Thirteen and 17 patients underwent surgery via a transcranial (T) and endonasal (E) route, respectively. No differences were seen in ASBQ, SNOT-22, and olfactory function between T and E, but in E were more full-time worker and less obesity. CSF leaks occurred in 15% of T and 29% of E (*p* = 0.43). Patients from group E had a superior visual outcome which was most pronounced in the visual field. The degree of new anterior and posterior pituitary gland deficiency after surgery and in the follow-up was lower in group E. The general and sinonasal quality of life and the olfactory function are equal in E and T. E is associated with a superior visual outcome, lower rates of diabetes insipidus, and lower rates of obesity, but has a higher risk for postoperative CSF leaks.

**Electronic supplementary material:**

The online version of this article (10.1007/s10143-020-01343-x) contains supplementary material, which is available to authorized users.

## Introduction

The treatment of patients with craniopharyngiomas has been a major challenge to all time of modern neurosurgery starting from Harvey Cushing [27, 29, 31, 35]. The main goals of treatment are tumor control and excellent functional outcome, including visual, pituitary, and hypothalamic function, but also a favorable neuropsychological outcome and quality of life. Both, the tumor growth and its respective (non-) surgical treatment can cause a disaster of functional outcome.

Since its evolution, the extended endoscopic endonasal approach has been a valuable alternative to transcranial corridors in the treatment of suprasellar craniopharyngiomas [14, 22, 33]. Studies addressing the comparison of transcranial and endonasal approaches to craniopharyngiomas have shown that the endonasal approach is associated with an improved visual outcome, but more postoperative CSF leaks. Assessment of quality of life in craniopharyngioma patients has been done separately to patients who underwent a transcranial approach or patients who underwent an endoscopic endonasal approach [26, 28]. A comparative analysis and the quantification of the olfactory function in those patients have not been done, yet.

The goal of the present study is to compare transcranial approaches and the endoscopic endonasal approach to suprasellar craniopharyngiomas with regard to quality of life and olfactory function.

## Methods

The study was approved by the local ethics board (BB155/17) and informed consent was obtained from all patients. All patients treated for a suprasellar craniopharyngioma in our department between 2001 and 2018 were included in the study. A comparative analysis was done between all patients who underwent surgery via a transcranial approach and all patients who underwent surgery via an endonasal route. All relevant patient characteristics as age at diagnosis, gender, clinical symptoms, perioperative nuances (extent of resection, anatomical preservation of pituitary stalk, complications), pre- and postoperative pituitary function, body mass index, and visual disturbances and adjuvant therapies were gathered from a prospectively maintained database. The extent of resection was determined by the intraoperative impression as well as the postoperative MRI.

### Visual outcome

Ophthalmological data were based on determination of visual acuity and visual field by a neuro-ophthalmologist. The outcome was divided into improved, stable, or deteriorated compared with the preoperative status. Furthermore, a quantitative analysis of visual acuity and visual field was done as previously described [19, 21]. To assess the visual acuity, the modified logMAR scale was used. To assess visual field deficits, an ordinal scale was used with the following score: 6 indicates normal visual field; 5, slight constriction; 4, loss of a single quadrant; 3, loss of 2 quadrants; 2, loss of 3 quadrants; 1, severe constriction; and 0, blindness [19, 21].

### Quality of life

During the last follow-up visit (12 months earliest after surgery), quality of life was assessed with the anterior skull base quality of life questionnaire (ASBQ) which has been developed and validated for quality of life measurements in patients suffering from skull base lesions [3, 9]. The ASBQ consists of 35 items with responses recorded on a 5-item Likert scale, ranging from 1 to 5 points per item. A higher score is indicating a higher quality of life. The ASBQ, furthermore, can be divided into 5 domains of life quality. These include physical function (questions 5, 16–20, and 31), vitality (questions 6–7, 21–22, 32, and 34–35), pain (questions 23–24 and 33), influence of emotion (questions 8 and 25–28), and specific symptoms (questions 9–15) [28].

Because the sinonasal quality of life is of particular relevance after endonasal surgical approaches, the SNOT-22 questionnaire was chosen as a further instrument to assess the quality of life in this study. The SNOT-22 questionnaire consists of 22 questions and responses are recorded on a 6-item Likert scale, ranging from 0 to 5 points per item. Total scores range from 0 to 110, with a higher score indicating a worse quality of life. The questionnaires were done by the patients in their native language (German). As further metric for the quality of life, the working status of patients and the body mass index were assessed.

### Olfactory function

During the last follow-up visit (12 months earliest after surgery), assessment of olfactory function was done. Olfactory function was assessed by the “Sniffin’ Sticks” test (Burghart Messtechnik GmbH, Wedel, Germany) for each nostril separately. The patient has to identify 12 different smells in a blinded fashion, whereby he can choose out of 4 possible options.

### Surgical procedures

#### Transcranial approach

**Fig. 1:**
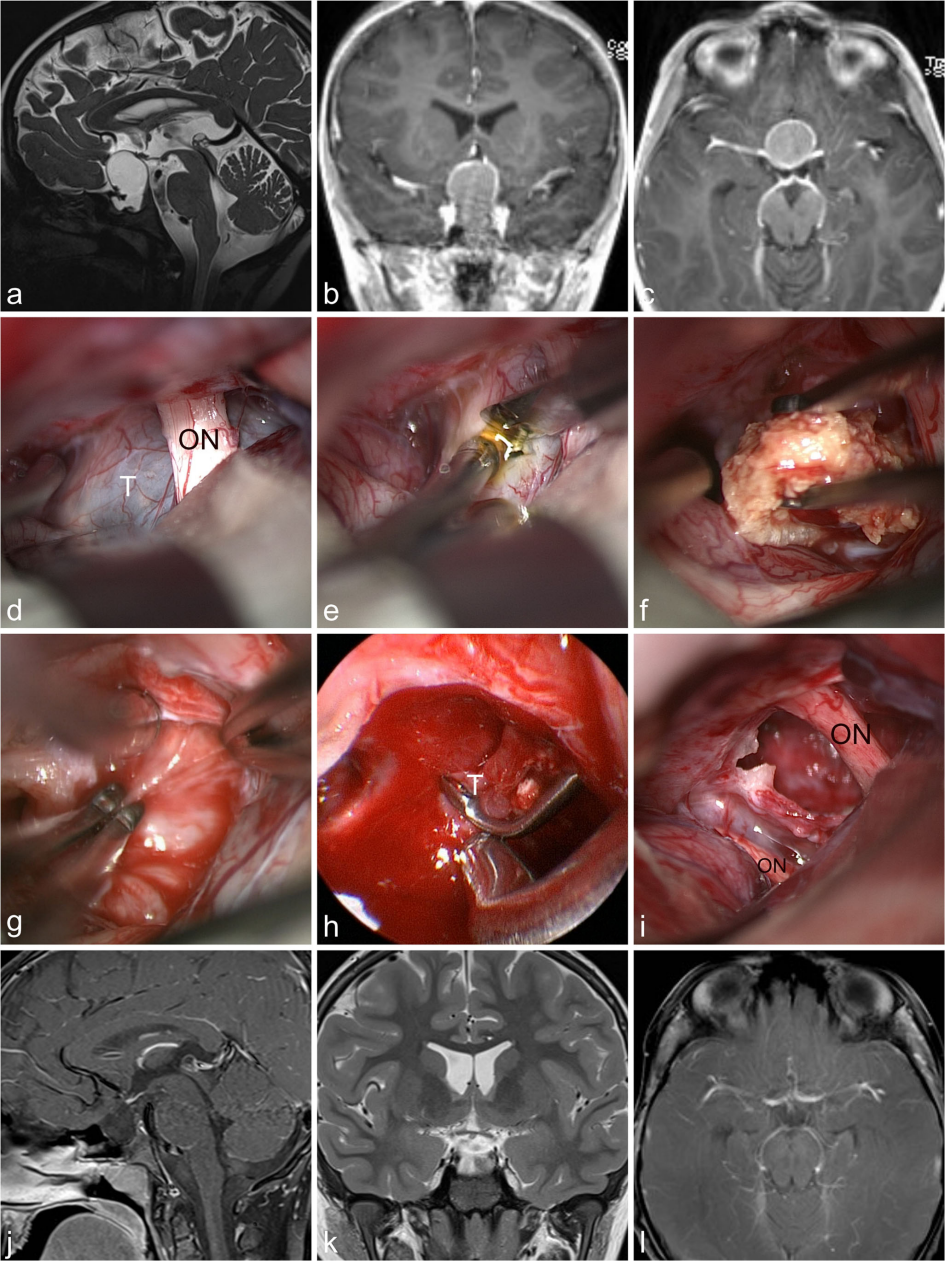
The 7-year-old boy presented with signs of hypopituitarism such as loss of weight, arrest of growth, impairment of his general condition, and fatigue. Since 4 weeks he had been complaining about headache and vomiting. The endocrinological evaluation demonstrated panhypopituitarism, but no obvious DI. **a**–**c** MR imaging revealed an intra-suprasellar circumferentially contrast enhancing cystic lesion which was highly suspicious of a craniopharyngioma. **d** The tumor (T) was approached in the interoptic window via a right-sided frontolateral approach exposing the right optic nerve (ON). **e** The tumor capsule was incised with a knife. **f** Calcified tumor parts were removed with tumor forceps. **g** The tumor was dissected using the bimanual traction-countertraction technique with two forceps. **h** Intrasellar tumor parts (T) which could not be visualized with the microscope were removed under endoscopic view of a 30° endoscope. **i** The final inspection showed the gross total tumor resection and intact optic nerves (ON**). j**–**l** MR imaging obtained 2 years after surgery showed no recurrence. The boy is doing well under full hormonal replacement therapy

The pterional and frontolateral approaches are our first choices in transcranial surgery for suprasellar craniopharyngiomas. All surgeries were done by the senior author (HWSS). After general anesthesia had been induced, the patient was placed supine in a three-pin fixation with the head in extension and rotated to the contralateral side in a way that the zygoma was the highest point. Skin incision was usually behind the hairline. After craniotomy a key step was to release CSF by opening the Sylvian fissure. Of utmost importance was the relation of the tumor to the optic chiasm and hypothalamus. The tumor was resected via the interoptic, optocarotid, or retrocarotid window. If the tumor had intraventricular extensions, a lamina terminalis approach was added. Endoscope assistance was used frequently to improve visualization and reduce manipulations of the optic nerves and chiasm. A case example is shown in Fig. 1 and video 1.

#### Endoscopic endonasal transtuberculum-transplanum approach

**Fig. 2:**
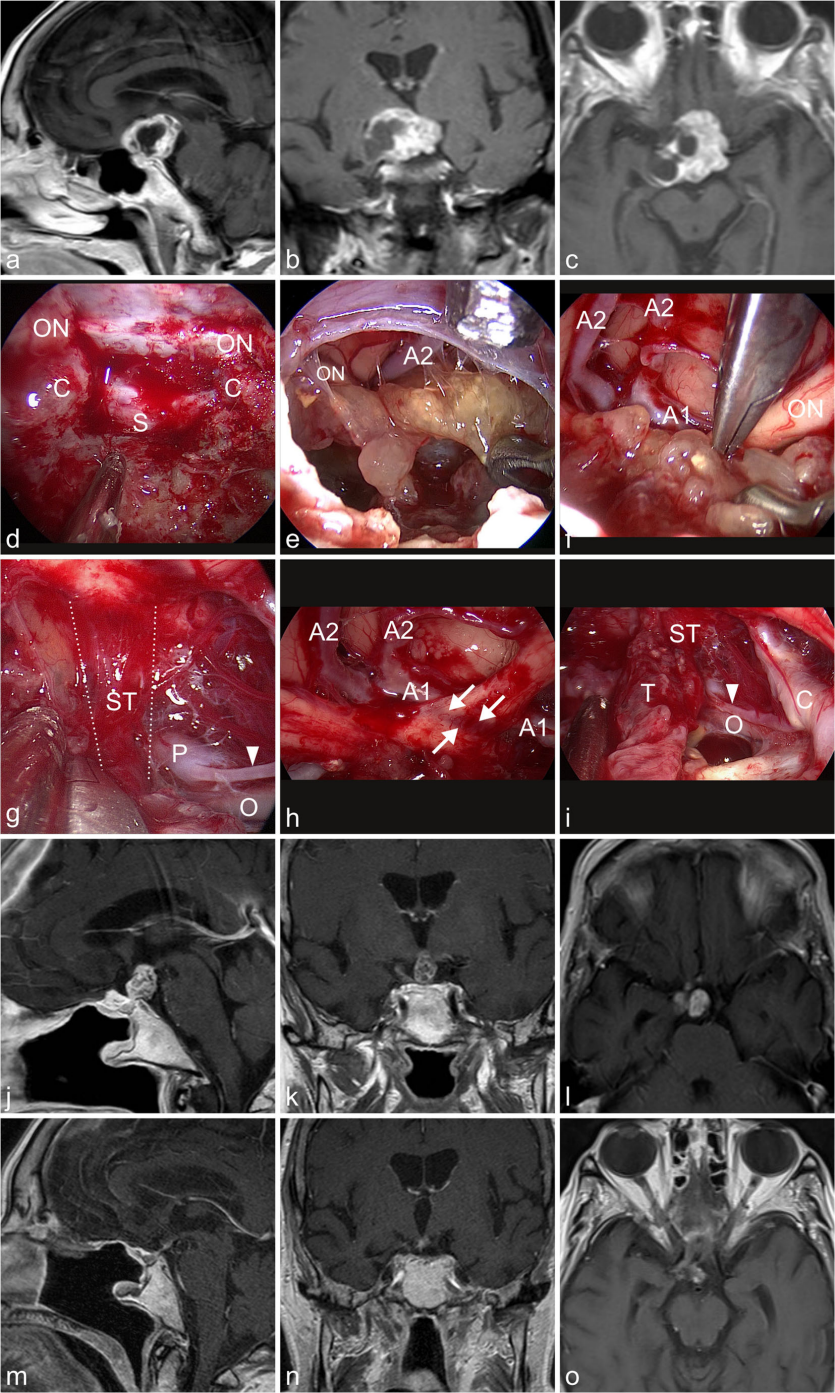
The 84-year-old lady presented with visual problems. The ophthalmological examination revealed a bilateral loss of visual acuity and bitemporal visual field deficits. The endocrinological evaluation demonstrated an intact pituitary function. **a**–**c** MR imaging revealed a suprasellar partially solid, partially cystic contrast enhancing lesion which was highly suspicious of a craniopharyngioma. **d** A transtuberculum-transplanum approach was performed. The protuberances of the optic nerves (ON), clinoidal carotid arteries (C), and sellar dura (S) are seen. **e** After debulking of the tumor, the superior surface of the tumor was exposed under view of a 30° endoscope. The A2 segment of the right anterior cerebral artery and the right optic nerve (ON) are seen. **f** The tumor was dissected from the optic chiasm using the bimanual traction-countertraction technique with two forceps (optic nerve (ON), A1- and A2-segments of the anterior cerebral arteries (A1, A2). **g** At the dorsal tumor surface, the infiltrated pituitary stalk (ST, in between the dashed line) is visualized. Oculomotor (O), P1-segment of the posterior cerebral artery (P), and posterior communicating artery (arrowhead) are seen. **h** After tumor resection severe grooving (arrows) within the left optic nerve caused by the tumor pressure against the nerve and the A1-segment of the anterior cerebral artery (A1 und A2) is seen. **i** The final inspection shows the tumor remnant (T) at the infiltrated pituitary stalk (ST), the posterior communicating artery (arrow head), the supraclinoidal carotid artery (C), and the oculomotor nerve (O). **j**–**l** MR imaging obtained 3 months after surgery showed the tumor residual at the pituitary stalk. **m**–**o** MR imaging obtained 22 months after fractionated stereotactic radiotherapy with 45 Gy revealed an impressive shrinking of the tumor. The patient is doing well under replacement therapy of hydrocortisone and thyroxine. The ophthalmological examination revealed a marked improvement of the visual field deficits

Our technique of endoscopic endonasal resection of craniopharyngiomas was described by our group previously [1]. All surgeries were performed by the senior author (HWSS). In 13 patients, the ENT surgeon (WH) acted as surgeon during the intranasal and sphenoid phase of the surgery. After general anesthesia had been induced, the patient was placed supine with elevated body (30°) in a three-pin fixation. Key steps of the nasal phase were the bilateral lateralization of the inferior and middle turbinate, harvesting a nasoseptal flap and removal of posterior parts of the nasal septum with generation of a reverse flap [11, 15]. The right middle turbinate was removed in 10 patients (59%), because the nasal space was narrow. The sphenoid phase started with removal of the sphenoid rostrum, followed by a posterior ethmoidectomy to expose the tuberculum sellae and planum sphenoidale sufficiently. The sphenoid mucosa was removed and bony septa were drilled. Thereafter, a transtuberculum-transplanum approach was created by drilling of the upper sellar floor, tuberculum sellae, and adjacent planum. Clinoidal carotids and proximal optic canals were usually unroofed. After coagulation of the superior intercavernous sinus, a V-shaped dural incision was performed. The diaphragma sellae was cut to visualize the pituitary stalk. The arachnoid was opened and the branches of the superior hypophyseal arteries were identified. The tumor was debulked and dissected from adjacent neurovascular structures. If no plane was identified between tumor and hypothalamus, the resection was stopped. An infiltrated stalk was sacrificed when the patient presented with panhypopituitarism. When the pituitary function was sufficient, the stalk was not cut. The skull base was closed with fat, fibrin glue, and nasoseptal flap. When the third ventricle was opened, a lumbar drainage was placed for 5 days. A case example is shown in Fig. 2 and video 2.

### Statistical analyses

Continuous data were analyzed with the Mann-Whitney *U* test. Nominal data were analyzed with the Fisher exact test. Statistically significant data were assumed with a *p* value ≤ 0.05.

## Results

### Patient cohort

Thirty consecutive patients who underwent surgery for a suprasellar craniopharyngioma in our department were included in the study. Thirteen patients (43%) had their surgery via a transcranial (group T) and *n* = 17 patients (57%) via an endoscopic endonasal approach (group E). Three patients (23%) of group T and 1 patient (6%) of group E were pediatric patients (*p* = 0.29), which accounts for the differences in mean age between both groups. Mean age was 31.2 years (range from 1 to 61 years) in group T and 48.5 years (range from 12 to 84 years) in group E (*p* = 0.04). Considering adult patients only, the mean age was 40 years in group T and 50 years in group E (*p* = 0.12). Group T consisted of 8 female and 5 male and group E consisted of 8 female and 9 male patients (*p* = 0.48). Twelve patients of group T and 16 patients of group E were primary tumors (*p* = 1). One patient of each group had initial surgery years ago in another hospital.

Leading clinical complaints prior to diagnosis in group T were headache (*n* = 7), ophthalmological symptoms (*n* = 5), hydrocephalus (*n* = 3), increase of body weight (*n* = 5), and Addisonian crisis (*n* = 1). Leading clinical complaints prior to diagnosis in group E were headache (*n* = 6), ophthalmological symptoms (*n* = 9), hydrocephalus (*n* = 2), increase of body weight (*n* = 1), and Addisonian crisis (*n* = 2). The mean body mass index prior to surgery was 24.6 (range from 14.1 to 34) in group T and 26.5 (range from 21 to 40.8) in group E (*p* = 0.59).

Mean follow-up was 136 months (range from 34 to 202 months) in group T and 56 months (range from 12 to 145 months) in group E (*p* = 0.002). Long-term follow-up was not available for 3 patients in each group (*p* = 1). In group T, 1 patient died in the perioperative period and 2 patients could not be contacted, since they are from foreign countries. In group E, 3 patients were treated recently and no conclusions about follow-up can be drawn so far. During follow-up 2 patients of each group had surgery for recurrent tumor growth (*p* = 1). Adjuvant radiotherapy was applied in group T because of tumor regrowth in one patient and as a planned procedure after subtotal tumor removal in one patient. Adjuvant radiotherapy was applied in group E because of tumor regrowth in 2 patients and as a planned procedure after subtotal tumor removal in 4 patients. In the last follow-up, no patient of both groups showed a tumor recurrence or progressive growth of a known remnant. These results are shown in Table 1.

**Table 1:** Characteristics of patients with a transcranial or endonasal approach to suprasellar craniopharyngiomas. *p* ≤ 0.05 is considered statistically significant. *NS* nonsignificant

		Total	Group transcranial		Group endonasal		*p* value
		*n* count	*n* count	%	*n* count	%	
No. of patients		30	13	43	17	57	
Age (years)	Mean	41	31.2		48.5		0.04
	Range	(1 … 84)	(1 … 61)		(12 … 84)		
Female		16	8	62	8	47	NS
Male		14	5	38	9	53	NS
Pediatric patients		4	3	23	1	6	NS
Initial diagnosis		28	12	92	16	94	NS
Follow-up	Mean (months)		136		56		0.002
	Range		(34 … 202)		(12 … 145)		
	Lost-to	6	3	23	3	18	NS
Repeat surgery	Total	4	2	15	2	12	NS
	Transcranial	4	2		2		
	Endonasal	0	0		0		
Adjuvant radiation	All indications	8	2	15	6	35.3	NS
	After STR	5	1		4		
	Tumor recurrence	3	1		2		

### Surgical procedures

The surgical approaches in group T included pterional/frontolateral, supraorbital, and interhemispheric precallosal in 9, 2, and 2 patients respectively. Four of these patients received a transventricular cyst evacuation in the months prior to surgery in another hospital. All surgical procedures in group T were endoscope-assisted. Every patient of group E got an endoscopic endonasal tumor resection. One patient received an endoscopic transventricular cyst evacuation 1 month prior to surgery.

The mean duration of surgery did differ between the transcranial and endonasal group (343 versus 419 min, respectively). However, the difference was mainly caused by one exceptionally long endonasal surgery (858 min) in a huge suprasellar-retroclival calcified lesion which required prolonged drilling of the skull base and time-consuming sharp dissection of the calcified tumor mass. If that case is excluded, the main surgical time was 393 min and not significantly different from the transcranial series. Gross total resection was achieved in 7 patients (54%) of group T and 10 patients (59%) of group E (*p* = 1). Due to adherences to the pituitary stalk, hypothalamus, optical pathway, or carotid artery, a subtotal resection only was performed in 5 patients (38%) of group T and 3 patients (18%) of group E (*p* = 0.44). A partial tumor removal was planned before surgery in 1 patient (8%) of group T and 4 patients (23%) of group E (*p* = 0.35). The pituitary stalk could be preserved in 5 patients of group T (39%) and 13 patients (76%) of group E, nearing statistical significance (*p* = 0.06). These results are shown in Table 2.

**Table 2:** Surgical characteristics of patients with a transcranial or endonasal approach to suprasellar craniopharyngiomas. *p* ≤ 0.05 is considered statistically significant. *NS* nonsignificant, *EOR* extent of resection, *GTR* gross total resection, *STR* subtotal resection, *CSF* cerebrospinal fluid

		Total	Group transcranial		Group endonasal		*p* value
		*n* count	*n* count	%	*n* count	%	
EOR	GTR	17	7	54	10	59	NS
	STR	8	5	38	3	18	NS
	Partial	5	1	8	4	23	NS
	Stalk preservation	18	5	39	13	76	0.06
Complications	All	17	5	39	11	65	0.14
	CSF leak	7	2	15	5	29	NS
	Perioperative death	1	1	8	0	0	NS
	Meningitis	2	0	0	2	12	NS
	Hydrocephalus	2	0	0	2	12	NS
	Other	4	2	15	2	12	NS

### Perioperative complications

In group T and group E, 5 and 12 complications occurred, respectively (*p* = 0.14). CSF leaks occurred in 2 patients of group T (15%) and 5 patients (29%) of group E (*p* = 0.43). Of note, 4 CSF leaks in group E occurred during the first 7 surgeries, which means that the CSF leak rate was 57% in the first 7 surgeries and 10% in the last 10 surgeries indicating the learning curve. Further complications in group T included 1 small cerebellar hemorrhage, one fusiform aneurysm at the carotid artery, and one perioperative death due to a lung arterial embolism 22 days after surgery. Further complications in group E included the persistence of hydrocephalus with consecutive VP-shunt placement in two patients (these patients already had a beginning hydrocephalus by the tumor itself before surgery), meningitis with consecutive antibiotic treatment in 2 patients, one minor lung embolism, and one re-bleeding in the surgical cavity with the need for redo surgery 3 days after operation. These results are shown in Table 2.

### Ophthalmological outcome

#### Visual acuity

Prior to surgery, 11 eyes of group T (42%) and 11 eyes of group E (39%) had a decreased visual acuity related to the tumor (*p* = 1). The median logMAR on the left eye was 0.1 in both groups (*p* = 0.88). The median logMAR on the right eye was 0.1 in group T and 0.2 in group E (*p* = 0.44).

Postoperatively, visual acuity was improved in 5 eyes (45%) of group T and 10 eyes (91%) of group E (*p* = 0.02) and was deteriorated in no eyes of group T but 1 eye (4%) of group E (*p* = 1). This deterioration was only slight (logMAR decrease of 0.1). The median logMAR was 0.1 on both sides in both groups (*p* = 0.74 and 0.87, respectively).

In the long-term follow-up, visual acuity was improved in 7 eyes (64%) of group T and 10 eyes (91%) of group E (*p* = 0.52) and was deteriorated in 2 eyes of group T (18%) and 1 eye (9%) of group E (*p* = 0.54). Of note, 10 eyes (38%) in group T were lost to long-term follow-up. The median logMAR on the left eye was 0 in group T and 0.1 in group E (*p* = 0.46). The median logMAR on the right eye was 0.05 in group T and 0 in group E (*p* = 0.69). These results are shown in Table 3.

**Table 3:** Visual outcome of patients with a transcranial or endonasal approach to suprasellar craniopharyngiomas. *p* ≤ 0.05 is considered statistically significant. *NS* nonsignificant

		Group transcranial		Group endonasal		*p* value
		*n* count	%	*n* count	%	
Visual acuity						
Pre-OP	Impairment (eyes)	11	42	11	39	NS
	logMAR left eye	0.1		0.1		NS
	logMAR right eye	0.1		0.2		NS
Post-OP	Improved to pre-OP	5	45	10	91	0.02
	Deteriorated to pre-OP	0	0	1	9	NS
	Lost to follow-up	2	8	0	0	NS
	logMAR left eye	0.1		0.1		NS
	logMAR right eye	0.1		0.1		NS
Follow-up	Improved to pre-OP	7	64	10	91	NS
	Deteriorated to pre-OP	2	18	1	9	NS
	Lost to follow-up	10	38	2	7	0.008
	logMAR left eye	0	0	0.1		NS
	logMAR right eye	0.05		0		NS
Visual field						
Pre-OP	Impairment (eyes)	16	62	15	54	NS
	Visual field score left eye	5		5,5		NS
	Visual field score right eye	4		4		NS
Post-OP	Improved to pre-OP	2	8	10	36	0.02
	Deteriorated to pre-OP	2	8	0	0	NS
	Lost to follow-up	2	8	0	0	NS
	Visual field score left eye	4,5		6		0.01
	Visual field score right eye	4		6		0.06
Follow-up	Improved to pre-OP	5	31	13	50	NS
	Deteriorated to pre-OP	2	12	0	0	0.14
	Lost to follow-up	10	38	2	7	0.008
	Visual field score left eye	6		6		NS
	Visual field score right eye	6		6		NS

In group T, no statistically significant difference in the logMAR scale could be obtained preoperative compared with postoperative or to long-term follow-up. In group E there was a statistically significant improvement on the logMAR scale of the right eye between preoperative and long-term follow-up (*p* = 0.01). These results are shown in Table 5.

#### Visual field

Prior to surgery, 16 eyes of group T (62%) and 15 eyes of group E (54%) had visual field deficits (*p* = 0.59). The median visual field score on the left eye was 5 in group T and 5.5 in group E (*p* = 0.51). The median visual field score on the right eye was 4 in both groups (*p* = 0.86).

Postoperatively, visual field deficits were improved in 2 eyes (8%) of group T and 10 eyes (36%) of group E (*p* = 0.02) and were deteriorated in 2 eyes (12%) of group T but no eye of group E (*p* = 0.23). The median visual field score on the left eye was 4.5 in group T and 6 in group E (*p* = 0.01). The median visual field score on the right eye was 4 in group T and 6 in group E (*p* = 0.06).

In the long-term follow-up, visual field deficits were improved in 5 eyes (31%) of group T and 13 eyes (50%) of group E (*p* = 0.34) and were deteriorated in 2 eyes (12%) of group T but no eye of group E (*p* = 0.14). Of note, 10 eyes (38%) in group T were lost to long-term follow-up. The median visual field score was 6 on both sides for both groups (*p* = 0.26 and 0.29, respectively). These results are shown in Table 3.

In group T, no statistically significant difference in the visual field score could be obtained preoperative compared with postoperative or to follow-up. In group E, there was a statistically significant improvement on the visual field score on both sides between preoperative compared with postoperative or long-term follow-up. These results are shown in Table 5.

### Pituitary function

Prior to surgery, 6 patients (46%) of group T and 4 patients (24%) of group E had a adrenocorticotropic, 7 patients of group T (54%) and 6 patients (35%) of group E had a thyreotropic, 3 patients (23%) of group T and 8 patients (47%) of group E had a somatotropic, and 5 patients (38%) of group T and 7 patients (41%) of group E had a gonadotropic insufficiency of the pituitary gland. Furthermore, 2 patients (15%) of group T and 4 patients (24%) of group E suffered from diabetes insipidus. There were no statistically significant differences in this comparison (Table 4).

**Table 4:** Pituitary outcome of patients with a transcranial or endonasal approach to suprasellar craniopharyngiomas. *p* ≤ 0.05 is considered statistically significant. *NS* nonsignificant

		Group transcranial		Group endonasal		*p* value
		*n* count	%	*n* count	**%**	
Preoperative	Adrenocorticotrope	6	46	4	24	NS
	Thyreotrope	7	54	6	35	NS
	Somatotrope	3	23	8	47	NS
	Gonadotrope	5	38	7	41	NS
	Diabetes insipidus	2	15	4	24	NS
Postoperative	Adrenocorticotrope	12	92	11	65	0.1
	Thyreotrope	12	92	9	53	0.04
	Somatotrope	9	69	11	65	NS
	Gonadotrope	11	85	10	59	NS
	Diabetes insipidus	8	62	9	53	NS
	Lost to follow-up	1	7	0	0	NS
Long-term follow-up	Adrenocorticotrope	9	69	9	53	NS
	Thyreotrope	9	69	5	29	0.06
	Somatotrope	10	77	12	71	NS
	Gonadotrope	10	77	11	65	NS
	Diabetes insipidus	8	62	7	41	NS
	Lost to follow-up	3	23	3	18	NS

Postoperatively and during long-term follow-up, the amount of deficiencies increased statistically significant in group T in all axes as well as in the rate of diabetes insipidus (Table 5). The amount of deficiencies increased in group E as well, but to a lesser degree as in group T. There was no significantly increased diabetes insipidus in group E comparing preoperative status with postoperative status or the long-term follow-up (*p* = 0.12 and 0.15, respectively). These results are shown in Table 5.

**Table 5:** Visual and pituitary outcome as well as body mass index of patients with a transcranial or endonasal approach to suprasellar craniopharyngiomas prior to surgery compared with postoperative and the follow-up. *p* ≤ 0.05 is considered statistically significant. *NS* nonsignificant

		Visual acuity		Visual field deficits		Body mass index	Pituitary function (impaired axes)				
		logMAR left	logMAR right	VFD-score left	VFD-score right		Adreno	Thyreo	Somato	Gonado	Diab. Ins.
Transcranial	Pre-OP	0.1	0.1	5	4	24.6	6	7	3	5	2
	Post-OP	0.1	0.1	4.5	4		12	12	9	11	8
	Follow-up	0	0.05	6	6	35.1	9	9	10	10	8
	*p* value pre vs. post	NS	NS	NS	NS		0.005	0.01	0.01	0.02	0.01
	*p* value pre vs. FU	NS	NS	0.15	NS	0.008	0.07	0.08	0.0004	0.003	0.003
Endonasal	Pre-OP	0.1	0.2	5,5	4	26.5	4	6	8	7	4
	Post-OP	0.1	0.1	6	6		11	9	11	10	9
	Follow-up	0.1	0	6	6	29.8	9	5	12	11	7
	*p* value pre vs. post	NS	NS	0.04	0.12		0.04	NS	NS	NS	0.12
	*p* value pre vs. FU	NS	0.01	0.01	0.03	0.14	0.03	NS	0.06	0.07	0.15

### Quality of life

#### Working status

The working status showed up with huge differences in between both groups. Of the *n* = 17 patients with endonasal tumor removal, 2 patients have been retired because of age already prior to diagnosis/ surgery, 3 patients are not available for long-term follow-up (working status not known), one patient is working half-days, and 7 patients are full-time worker. Four patients were close to the (German) age of retirement at the time-point of diagnosis/ surgery and went into age-related retirement after surgery. In this group was one child which finished normal school and is now doing a qualification. Only one adult patient (36 years old) has not enough power to work at all.

Of the *n* = 13 patients with transcranial tumor removal, 3 patients have been retired because of age already prior to diagnosis/ surgery, 3 patients are not available for long-term follow-up (working status not known in 2, one died perioperatively), and 2 patients are full-time worker (one of them in a company for disabled persons). Four patients never did work after surgery; one of them is in a nursing home due to alcoholism. One child of this group finished a lower school education, but is now without job (nearly blind).

Summarized, only 2 patients (15%) of group T are working in full-time jobs, but one of them in a business for disabled persons. Seven patients (41%) of group E are working in full-time jobs.

#### Body mass index

The mean body mass index at the last follow-up was 35.1 (range from 28.5 to 43.5) in group T and 29.8 (range from 24 to 39.1) in group E (*p* = 0.05). The difference of BMI from prior to surgery to the last follow-up was statistically significant (*p* = 0.008) in group T, but not in group E (*p* = 0.14).

#### ASBQ test

The median ASBQ score was 3.9 (range from 2.9 to 4.8) in group T and 4.6 (range from 3.4 to 5) in group E which did not reach statistical significance (*p* = 0.14). Actually, both groups did not show any differences in the dimensions “physical function,” “vitality,” and “specific symptoms” (*p* = 0.39, *p* = 0.21, *p* = 0.8, respectively). The median score in the dimension “influence of emotion” was 3.8 (range from 2.2 to 4.8) in group T and 4.6 (range from 2.4 to 5) in group E (*p* = 0.13). The median score in the dimension “pain” was 3 (range from 1 to 5) in group T and 4.7 (range from 2.3 to 5) in group E (*p* = 0.07). However, this difference is mostly due to the fact that 2 patients in group T suffered from severe back pain during follow-up. Furthermore, there was no statistically significant difference between patients who got radiation therapy and those who did not (*p* = 0.79).

### SNOT

The total SNOT score was 17 (range from 0 to 52) in group T and 7 (range from 0 to 42) in group E (*p* = 0.39). Furthermore, there was no statistically significant difference between patients who got radiation therapy and those who did not (*p* = 0.77).

### Olfactory function

The median score in the smell screening test on the left side was 7 points (range from 4 to 11) in group T and 9 points (range from 2 to 11) in group E (*p* = 0.98). The median score in the smell screening test on the right side was 7 points (range from 2 to 12) in group T and 8 points (range from 2 to 12) in group E (*p* = 0.81).

The median score in the Sniffin’ Sticks test on the left side was 7 points (range from 1 to 9) in patients who received radiotherapy and 10 points (range from 2 to 11) in patients who received no radiotherapy (*p* = 0.09). The median score in the Sniffin’ Sticks test on the right side was 3 points (range from 1 to 10) in patients who received radiotherapy and 10 points (range from 3 to 12) in patients who received no radiotherapy (*p* = 0.01). These results are shown in Table 6.

**Table 6:** Quality of life of patients with a transcranial or endonasal approach to suprasellar craniopharyngiomas. *p* ≤ 0.05 is considered statistically significant. *NS* nonsignificant

		Group T	Group E	*p* value
		*n* count	*n* count	
Long-term follow-up				
Working status	Full-time worker	2	7	0.1
ASBQ	(Median score)	3.9	4.6	0.14
SNOT-22	(Median score)	17	7	NS
Body mass index	(Mean)	35.1	29.8	0.05
Olfaction	Left side	7	9	NS
	Right side	7	8	NS

There was no difference between left and right nostril neither in all patients who underwent endonasal surgery nor considering only those with resection of the middle turbinate on the right side (*p* = 0.95).

## Discussion

### Summary of the main results

The present study reveals no difference in the quality of life in suprasellar craniopharyngioma patients who underwent surgery via a transcranial or endonasal route. Furthermore, the sinonasal quality of life and the olfactory outcome did not differ between both groups. The endonasal approach is accompanied with an improved visual outcome, but more perioperative complications as CSF leaks. Furthermore, the endonasal approach is associated with lower rates of diabetes insipidus, obesity, and a higher rate of full-time worker during follow-up.

### Limitations of the study

The striking limitation of the present study is the low number of patients. To obtain robust results to data regarding quality of life in such a complex disease like craniopharyngioma, multivariate analysis would have been necessary. However, this makes no sense in such small group sizes. Nevertheless, craniopharyngiomas are a rather rare disease and most series have a magnitude of size like we present in our study [7, 22, 25, 33]. A solution for this problem would be a large multicenter study, as was done by Little et al. with regard to the comparison of endoscopic versus microsurgical endonasal approaches to pituitary lesions [18]. Both groups of the present study are comparable in most parameters, but differ with regard to patient age and the length of follow-up. The difference in patient age is attributed to the fact that there is a slight but statistically not significant difference in the amount of pediatric patients. However, because of the low overall number of patients, we did not want to exclude the pediatric patients. There was only one recurrent case in every group. Thus, this is well balanced and should not bias the results. The difference in primary and revision cases was much more pronounced in other studies [14, 16]. The difference in follow-up is attributed to the fact that most of the transcranial procedures were done from 2001 to 2009 and most of the endonasal procedures were done from 2009 to 2018.

### Long-term quality of life and olfactory function

Besides conventional outcome parameter like extent of resection, postoperative neurological deficits, and overall survival, patients’ quality of life is of increasing interest after neurosurgical procedures. Quality of life describes the patients’ perception of well-being and is reported by the patients themselves. Although it is known that craniopharyngioma patients have an impaired quality of life after surgery [5], to date no comparative study has been done to the quality of life comparing transcranial and endonasal approaches to suprasellar craniopharyngiomas.

We have seen a slight trend of a better ASBQ outcome in the long-term follow-up in craniopharyngioma patients with an endonasal approach. However, this difference is mostly due to the fact that 2 patients in group T suffered from severe back pain during follow-up and is not related to the craniopharyngioma surgery itself. It is a major drawback of quality of life questionnaires that they capture quality of life changes which might be related to comorbidities instead of the disease which is examined [28]. In our point of view, the distribution of full-time worker in between the groups is a convincing argument for an improved outcome after endonasal approaches to suprasellar craniopharyngiomas. Patients with an endonasal approach do not have an inferior sinonasal quality of life measured by the SNOT-22 which is an important finding of the present study.

The ASBQ and SNOT-22 questionnaire are validated for lesions in the anterior skull base and were already applied to craniopharyngioma patients in another study [28]. The mean ASBQ in group E in our study was 4.6, which is slightly higher than that reported by Patel et al. in 31 endonasal approaches to craniopharyngiomas (ASBQ score 3.4) [28]. Group E in our study had a mean SNOT-22 score of 7 points which is lower than that reported by Patel et al. in 31 endonasal approaches to craniopharyngiomas (SNOT-22 score 19.6) [28].

The olfactory function had not been addressed in the comparison of transcranial to endonasal skull base approaches so far. Soyka et al. have noticed a significantly reduced olfactory outcome after nasoseptal flap reconstruction on the flap donor side compared with the opposite side [32]. We did not observe any differences between both sides in our endonasal group. That might be related to our flap harvesting technique which leaves 1 cm of septal mucosa at the skull base on both sides and to the preservation of both superior turbinates. No difference in the olfactory outcome between the left and right nostril could be obtained in patients with resection of the middle turbinate on the right side. This finding indicates that the resection has no influence on the olfactory outcome as shown previously [20].

Comparing absolute Sniffin’ Sticks results with other studies needs to be done with caution, because several different Sniffin’ Sticks tests exist on the market. We used a set of 12 odors to test which was done by other groups as well [10, 12]. Other groups used a set of 16 odors to test which leads inevitably to higher test results in a comparable patient population [4, 13, 23].

A further critical factor of the long-term quality of life in craniopharyngioma patients is the excessive obesity related to hypothalamic involvement of the tumor/ hypothalamic injury by the treatment. [6, 30] In the present study, it could be shown that the rate of obesity is decreased in endonasal approaches to craniopharyngiomas which could be confirmed by a recent study. [34]

### Visual outcome

Studies comparing transcranial and endonasal approaches to craniopharyngiomas show tremendous superiority of the endonasal approach with regard to the visual outcome. Improvement rates of 63% and 89% after endonasal resection stand against 25% or even 0% after transcranial resection [22, 33]. For the first time we used a quantitative approach for visual acuity and visual field deficits per eye rather than a qualitative statement per patient. We could show that especially the visual field improves highly significantly after endonasal resection of suprasellar craniopharyngiomas.

Nevertheless, even the patients in the transcranial group of our study showed an improvement of visual acuity (44%) and visual field deficits (31%) during follow-up. Another study has shown an improvement of the visual function in 70% of craniopharyngioma patients after the supraorbital keyhole approach [2]. Other studies found a higher rate of visual deterioration after transcranial approaches to suprasellar craniopharyngiomas [16, 33]. The key of a good visual outcome in surgery for anterior skull base lesions is the preservation of the superior hypophyseal arteries and avoiding manipulations of the optic nerves. Endoscope assistance helps in reducing the manipulations of the optic apparatus while using transcranial routes [2, 21] and might explain the better results compared with other microsurgical transcranial series. However, transcranial approaches still require dissection through narrow surgical windows, such as the interoptic, opticocarotid, or retrocarotid window. Additionally, most craniopharyngiomas cause a prefixed chiasm which narrows the windows even more. Therefore, there is no doubt that the endonasal approach is the far better choice for the resection of midline suprasellar craniopharyngiomas. It allows an early identification of the pituitary stalk and branches of the superior hypophyseal arteries. The tumor can be dissected without retraction of the optic nerves and chiasm.

### Pituitary outcome

The risk to develop a pituitary insufficiency, at least partial, is very high in the postoperative period and in the long-term follow-up after craniopharyngioma surgery—independent of the surgical approach [22]. Although both groups in the present study did not show any significant differences in the individual anterior or posterior pituitary axes, the deterioration in the postoperative period and during long-term follow-up was more pronounced in the transcranial resection group, as shown in Table 5. All axes in this group experienced a significant deterioration, whereas in the endonasal resection group the differences reached only in part statistical significance. Most studies are not addressing the single anterior pituitary lobe hormone axes, but rather make statements like “total” or “partial” anterior pituitary lobe deficiency [14, 16, 22, 25, 33]. Thus, their results are not comparable to our single-axis analysis.

The rate of diabetes insipidus (DI) was 41% in the endonasal resection group and 62% in the transcranial resection group which is in accordance with other studies [14, 33]. Both the rate of DI and the missing difference between the two approaches are observed by Wannemühler [33]. However, other studies could show a lower rate of permanent DI in endonasal series [14, 16, 17]. Interestingly, 2 patients of the endonasal group of our study did recover from postoperative diabetes insipidus, but no patient in the transcranial group. The slightly better endocrinological results in the endonasal group of our study might be associated to the fact that the pituitary stalk can be seen early during surgery and by that preserved more easily. However, even the preservation of the pituitary stalk is no guarantee for a favorable pituitary outcome [24].

### Philosophy of surgical radicality and adjuvant therapy

Craniopharyngiomas often adhere to critical neurovascular structures such as the pituitary stalk, optical apparatus, carotid artery, and hypothalamus. An aggressive surgical approach often leads to gross total resection, but the risk for functional deficits increases as well. A defensive surgical approach with significant tumor remnant leads to a higher rate of recurrence and the need for adjuvant therapy.

Prior to surgery we usually discuss different options with the patient. The main question is if the pituitary stalk should be preserved, even if a gross total resection would not be possible in this situation. Our philosophy has changed over the years in regard to this. In earlier years, we often did a gross total resection including pituitary stalk resection. Seeing the high rates of diabetes insipidus, we changed our philosophy. If the patient presents with pituitary insufficiency and DI, we sacrifice the stalk. If the patient is endocrinologically intact and the tumor can be dissected with preservation of the stalk, a gross total resection is performed. However, if the tumor resection would result in destruction of the stalk, we do a subtotal resection and add a fractionated stereotactic radiotherapy (30 × 1.8 Gy = 54 Gy) 3 months after surgery. That is for sure the main reason why we preserved the pituitary stalk more often in the endonasal group. Komotar et al. found a significantly higher degree of gross total resection in the endonasal approach [16]. However, in our point of view this is more surgical philosophy than “can be achieved.” If set as the goal, gross total resection is possible via both approaches.

### Skull base reconstruction

Although the nasoseptal flap is a valuable technique to cover large skull base defects, rates of CSF leakages are often still high in extended endonasal surgery, as shown by our series as well. Our overall CSF leakage rate was 35% in the endonasal group. In two patients who presented with tumor-related hydrocephalus, even shunting was required to stop the leakage. After 1 year the shunts were removed without the need of any other CSF diverting procedure during follow-up. However, considering only the last 10 surgeries, it dropped to 10% which is the rate what most other groups have shown for endonasal approaches to suprasellar craniopharyngioma [14, 16, 33]. However, smaller rates of CSF leaks are reported as well [8]. There is clearly a learning curve in skull base reconstruction. Some of our initial flaps might have been a little bit too small. Other groups have shown fewer CSF leaks when using the gasket-seal closure [8]. However, they experienced more visual deterioration which might be related to the closure technique which uses autologous fascia which was countersunk with a rigid buttress. We do not use rigid materials for closure because of the close neighborhood of the optic nerves. In the last 10 cases, we used fat to fill the dural defect and fixed it with fibrin glue. Then, the nasoseptal flap was placed over it in a manner that it overlaps the margin of the bony defect by app. 1 cm. The flap was fixed with fibrin glue as well. After placing Surgicell (© Ethicon, USA) and gel foam on the flap, 3 tamponades were inserted into each nostril to keep the flap in correct position. If the third ventricle was opened, a lumbar drain was placed for 5 days.

### Sinonasal quality of life

Another interesting result of the present study is that the sinonasal quality of life is not inferior after an endonasal approach during follow-up. In extended endonasal surgeries, we usually combine the procedure with the ENT surgeons, because they handle the nasal mucosa like a neurosurgeon the brain.

## Conclusion

The general and sinonasal quality of life and the olfactory function are equal in the endonasal and transcranial approach to suprasellar craniopharyngiomas. The endonasal approach is associated with a superior visual outcome, lower rates of diabetes insipidus, and lower rates of obesity, but has a higher risk for postoperative CSF leaks.

## Electronic supplementary material

ESM 1 (MP4 117,363 kb)

ESM 2 (MP4 178,792 kb)
